# Single‐cell transcriptomic landscape reveals distinct tumourigenesis and immune microenvironments in secondary radiation‐exposed rectal cancer

**DOI:** 10.1002/ctm2.1659

**Published:** 2024-04-17

**Authors:** Xu Guan, Xiaoman Bi, Ran Wei, Zhixun Zhao, Zhao Lu, Zheng Jiang, Xishan Wang, Deng Wu

**Affiliations:** ^1^ Department of Colorectal Surgery National Cancer Center/National Clinical Research Center for Cancer/Cancer Hospital, Chinese Academy of Medical Sciences and Peking Union Medical College Beijing China; ^2^ Department of Colorectal Surgery Shanxi Province Cancer Hospital/ Shanxi Hospital Affiliated to Cancer Hospital, Chinese Academy of Medical Sciences/ Cancer Hospital Affiliated to Shanxi Medical University Taiyuan China; ^3^ College of Biomedical Information and Engineering Hainan Medical University Haikou China; ^4^ Department of Gastrointestinal Surgery the First Affiliated Hospital of Sun Yat‐Sen University Guangzhou China; ^5^ Department of Gastrointestinal Surgery Zhongnan Hospital of Wuhan University Wuhan China; ^6^ School of Life Sciences, Faculty of Science The Chinese University of Hong Kong Hong Kong China

Dear Editor,

Radiation therapy is a common treatment option for pelvic cancer patients^1^ and can induce secondary radiation‐exposed rectal cancers (SRCs).^2^ However, compared with those of primary rectal cancers (PRCs), the molecular characteristics and the cause of SRCs are not fully understood.^3^ By sequencing the single‐cell transcriptome to reveal the cell type‐specific profile, this study revealed an augmented copy number variation (CNV) profile and a distinct immune microenvironment in SRCs.

By comprehensively evaluating the tumour types that occurred with the therapeutic ionising radiation and the short time, compared to the evolution of PRCs, a total of 18 SRCs were diagnosed (Table S1). Two classical SRCs and two PRCs as well as their normal tissues were sequenced at single‐cell resolution (Figure S1A, Table S1), resulting in 16 334 cells (Figure S1B) from 10 broad cell types, including epithelial cells, endothelial cells, fibroblasts, T cells, plasma B cells, naïve B cells, mast cells, macrophages, neutrophils and MKI67 progenitor cells (Figure 1A), according to their validated markers^4^, ^5^ (Figures 1B,C and S1B,C). t‐Distributed stochastic neighbour embedding (t‐SNE) analysis suggested that the major difference between normal cells, PRCs and SRCs was derived from epithelial cells (Figure 1D,E) and neutrophils (Figure 1D–F). A total of 2297 unique differentially expressed genes (DEGs) was identified (Figure 1G, Table S2) between tumour and normal tissues. A direct comparison of epithelial cells between PRCs and SRCs revealed that tumour‐activated pathways, such as DNA repair, apoptosis inhibition and mitosis, were significantly enhanced in SRCs, while oxidative phosphorylation was upregulated in PRCs (Figures 1H and S1D).

**FIGURE 1:**
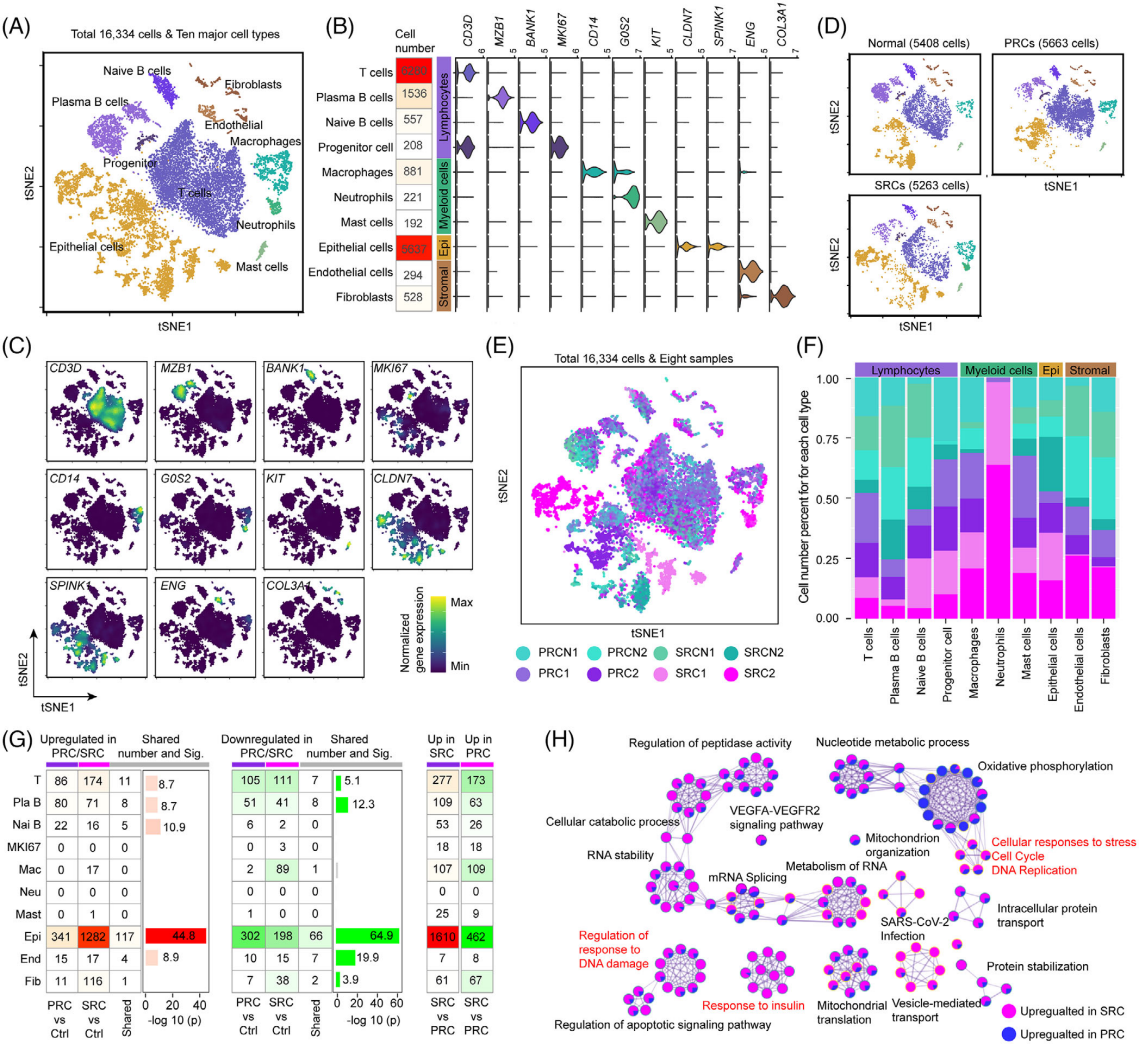
Cellular composition of primary and second rectal cancers and their global variation. (A) t‐Distributed stochastic neighbour embedding (t‐SNE) visualisation of 16 334 cells coloured according to their corresponding cell types. (B) Violin plots of cell type‐specific markers and the cell number statistics for each cell type. C. t‐SNE plots for the expression of cell type‐specific markers. (D) t‐SNE visualisation of samples from normal, primary rectal cancer (PRC) and second rectal cancer (SRC) patients. The cells are coloured according to their cell type. (E) t‐SNE visualisation of samples. (F) Cell number proportion for each cell type between samples. (G) Comparison of the number of differentially expressed genes (DEGs) between the tumour and normal samples (left two panels) and SRCs and PRCs (right panel). (H) Functional enrichment network for the DEGs between PRCs and SRCs from their epithelial cells. The blue nodes represent the enrichment results of the upregulated genes in epithelial cells from SRCs (*n* = 1610 genes), and the pink nodes represent the enrichment results of the upregulated genes in epithelial cells from PRCs (*n* = 452 genes).

A total of 18 subclusters was obtained from epithelial cells (Figure 2A), six subclusters were initially treated as candidate normal cells (Figures 2B and S2A), and another 12 subclusters were defined as potential tumour cells because they originated from one tumour sample (Figures 2B and S2A). DNA CNV analysis confirmed that Cluster 0 and 10 displayed large‐scale genomic rearrangements, including gains of chr 8q, chr 16q and chr 20p (Figures S2B and 2C). Cluster 13, which originated from another SRC patient, shared a gain of chr 8q with Cluster 0 and 10. The PRC‐related clusters exhibiting a substantial number of CNV were Clusters 3 and 15. Overall, we defined Cluster 0, 10 and 13 as the SRC tumour cells, which displayed the highest CNV score (Figure S2C) and the highest differentiation capacity (Figures 2D and S2C) regardless of the dimensionality reduction method (Figure S2D,E). The specific highly expressed genes from the SRC tumour cells were validated in additional SRC samples (Figure S2G–I) further confirming the common features of the SRC malignant cells.

**FIGURE 2:**
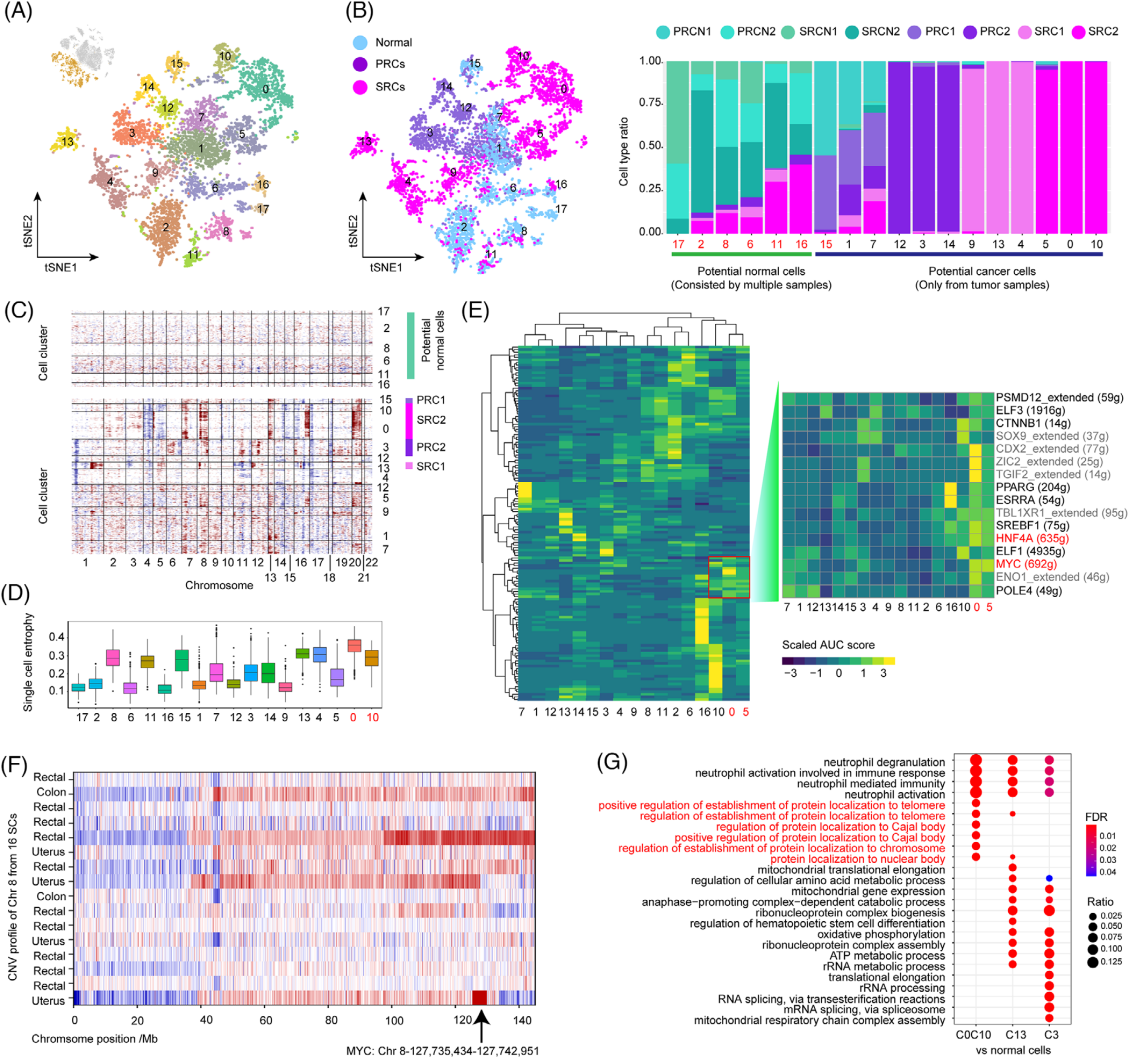
The landscape of rectal epithelial cells. (A) t‐SNE visualisation of reclustered epithelial cells across normal and tumour tissues (the inset shows included cells). (B) Left, t‐SNE visualisation of epithelial cells between SRCs and PRCs. Right, the relative proportions of normal and cancer samples in each subcluster. The first six subclusters were treated as potential normal cells since they were shared by multiple samples, while the last 12 subclusters were considered as potential tumour cells as they were derived from a single tumour sample. (C) Single‐cell copy number variation profiler for each subcluster. (D) Differentiation ability of each cluster predicted by single‐cell entropy. (E) Identification of transcription factors in each cluster. The activated transcription factors in Cluster 0 were amplified. (F). Heatmap displaying the copy number gain (red) and loss (blue) in chr 8 of the genomes of 16 secondary cancers. (G). Functional enrichment of upregulated genes, compared to the normal epithelial cells in Clusters 0, 10, 13 and 3.

The amplification of chr 8q led to increased gene expression of the tumour driver gene *MYC* (Figure 2E), an activated transcription factor across tumour cells (Figure 2E), which has been observed in secondary angiosarcoma^6^ and radiogenic adenocarcinoma.^7^ The CNV profile of the 18 additional SRCs determined by whole‐genome sequencing further validated the gain of chr 8q among the SRCs (Figure 2F). These additional CNVs might contribute to telomere maintenance among these tumour cells (Figure 2G).

We next reclustered the immune cells and identified 16 T‐cell subclusters (Figure 3A) from CD4^+^, CD8^+^ and natural killer T cells (Figure S3A–C). CD4^+^ clusters contained T helper 1 cells (*IL7R*/C1, C13), T helper 17 cells (*IL17A*/C7), activated T cells (*TNFRSF*4/C4), naïve/center memory T cells (*CCR7*/C5), two types of regulatory T cells (*CTLA4*/C9, *FOXP3* /C15) and another unsigned subcluster marked by *GPM6B*. Among the eight subclusters of CD8^+^ T cells, two were CD8^+^ effector T cells (*GZMK*/C0, GZMB/C2), one highly expressed *CXCL13* (C3), and the other was activated CD8^+^ T cells (*CRTAM*/C6). The remaining three clusters belong to natural killer cells (*KLRC1*/C10, *XCL1*/C11 and *FCGR3A*/C14).

**FIGURE 3:**
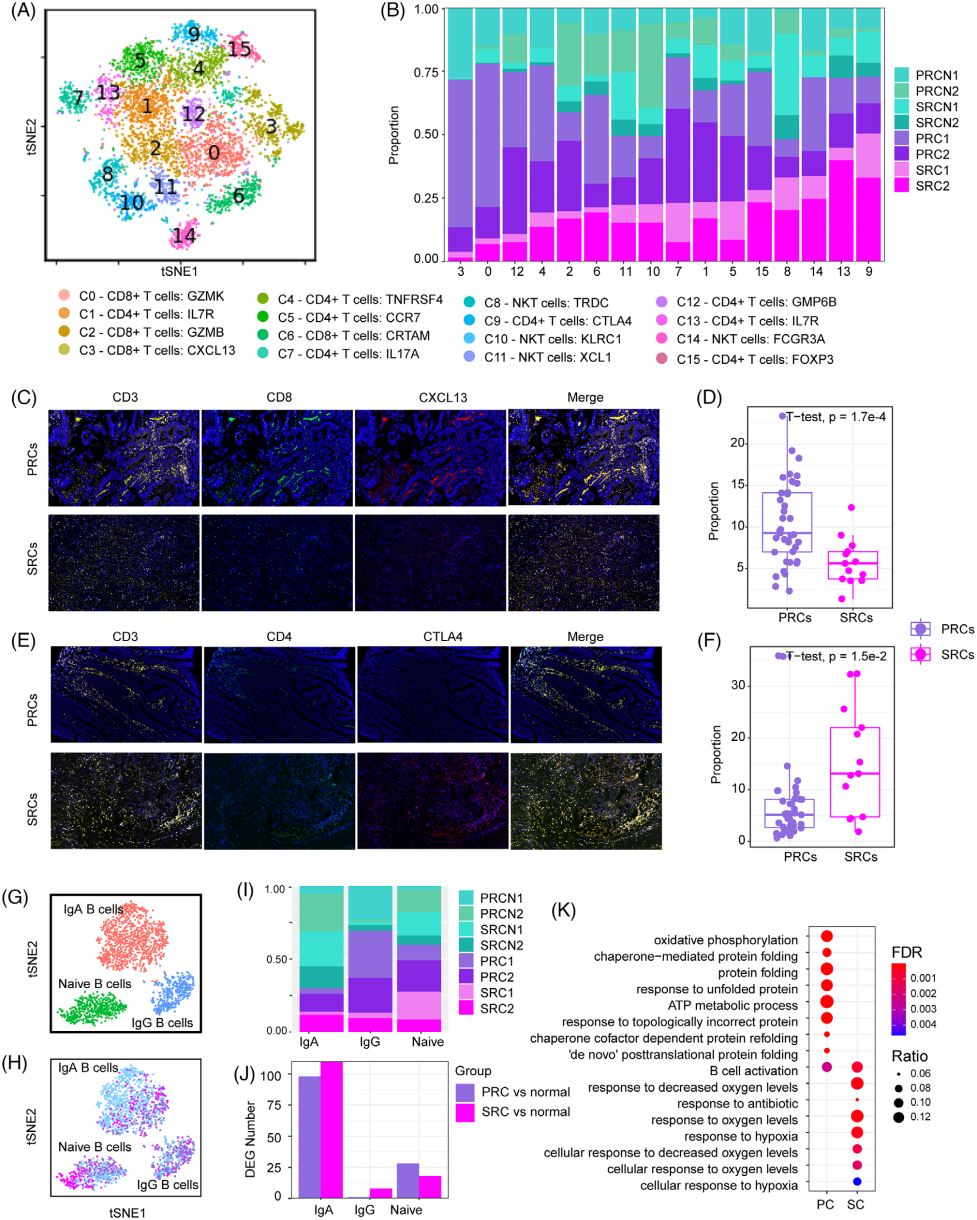
Lymphocyte landscape of PRCs and SRCs. (A) T cells were reclustered into 16 subclusters. (B) Relative proportion of sample composition in each subcluster. (C) Immunofluorescence staining of CD8+CXCL13+ T cells from normal cells, PRCs and SRCs. (D) Quantification of CD4+ CXCL13+ T cells based on whole‐slide scans of 39 PRC and 13 SRC specimens from (C). (E) Immunofluorescence staining of CD4+ CTLA4+ T cells from normal cells, PRCs and SRCs. (F) Quantification of CD4+CTLA4+ T cells based on whole‐slide scans of 39 PRCs and 13 SRCs specimens from (E). (G) t‐SNE visualisation of reclustered B cells; cells were coloured according to cluster and tumour type (H). (I) Proportion of B cells in each sample. (J) Number of DEGs between PRCs/SRCs and normal cells in each subtype of B cells. (K) Biological process enrichment of DEGs from IgA B cells.

We found that the number of CXCL13^+^ CD8^+^ T cells (C3) increased in the PRCs (Figure 3B) and CTLA4^+^ CD4^+^ T cells (C9) accumulated in the SRCs (Figure 3B). Analysis of tissue sections stained for CXCL13 (Figure 3C) and CTLA4 (Figure 3E) in an additional 39 PRCs and 13 SRCs further validated a significantly lower percentage of CXCL13^+^ CD8^+^ T cells in the PRCs (Figure 3D) and a greater percentage of CTLA4^+^ CD4^+^ T cells in SRCs (Figure 3F) than in the normal tissues.

In addition to the above divergent T‐cell subtype shift between PRCs and SRCs, cell proliferation‐associated genes *FOS* and *JUN* are differentially regulated between PRC and SRCs (Figure S3D,E), and the expression of cell migration‐associated genes, including *TMSB10* and *TMSB4X*, decreased in SRCs but did not change in PRCs (Figure S3D,E). The expression of heat shock response‐associated genes^8^ increased in both PRCs and SRCs (Figure S3D,E).

Naïve B cells, IgA B cells and IgG B cells were recognised among the B cells (Figure 3G,H). IgA B cells were the most significantly affected in both the PRCs and SRCs (Figure 3I) and had the most DEGs in both PRCs and SRCs (Figure 3J). Oxidative phosphorylation and the ATP metabolic process were enriched in PRCs, and hypoxia response was overrepresented in SRCs (Figure 3K).

Among the myeloid cell subtypes (Figures 4A and S4A), neutrophils were observed in SRCs (Figure 4B), and all subtypes of macrophages increased in both PRCs and SRCs (Figure S4B). A greater number of DEGs were found in M2‐like (tumour‐like) macrophages between SRCs and normal tissue (Figure 4C).

**FIGURE 4:**
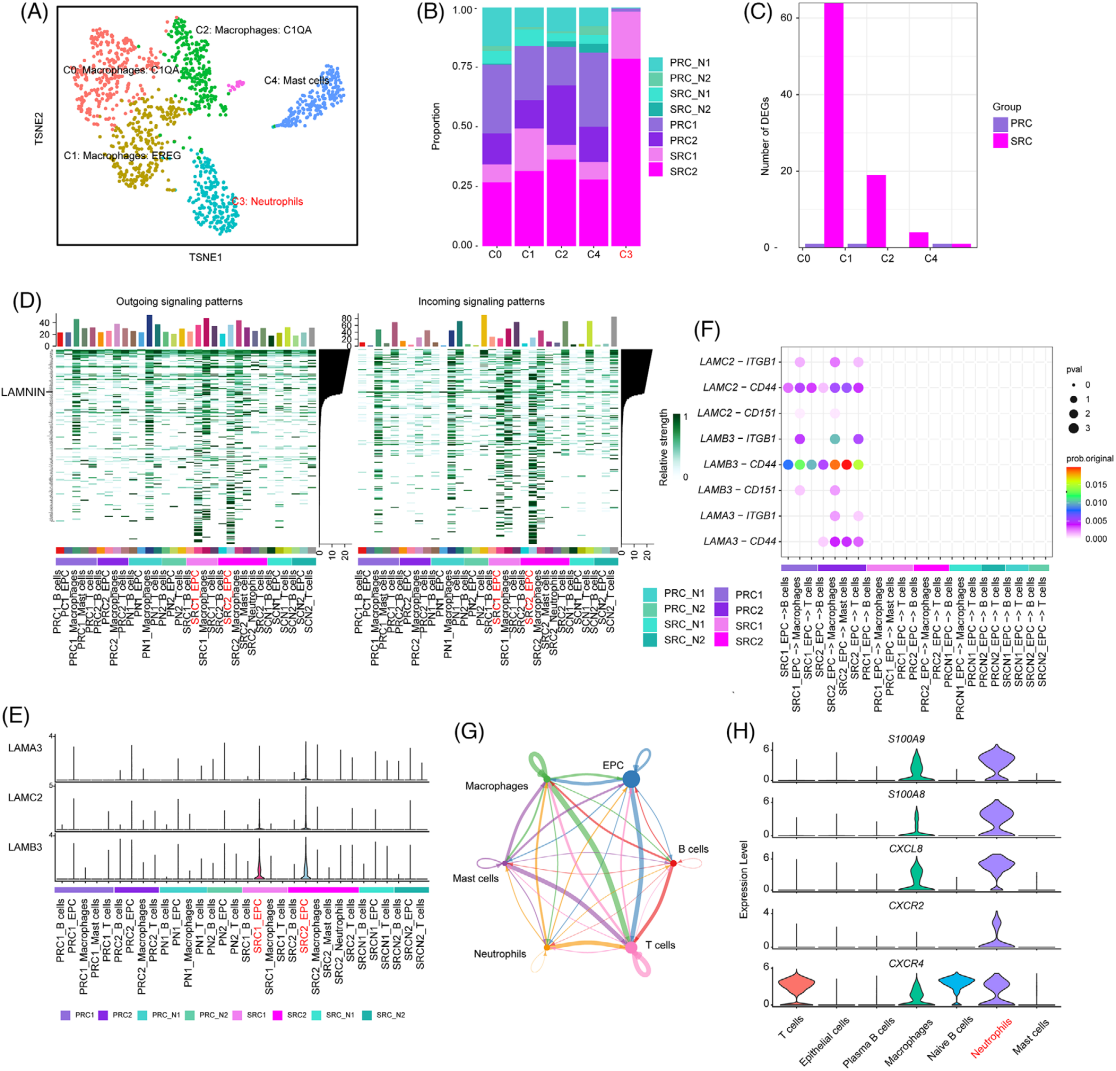
Rectal myeloid landscape and cell communication variation in SRCs. (A) Reclustered myeloid cells were divided into five subclusters, including three different macrophage types, neutrophils and mast cells. (B) Relative proportion of the sample composition in each myeloid cell subcluster. (C) Number of DEGs found in each subtype of B cells. (D) Statistics of outgoing and incoming signalling patterns for each cell type split by single samples. (E) High expression of genes associated with lamin and calmodulin in the SRC. (F) Lamnin‐associated cell–cell crosstalk was observed only in SRCs. (G) Neutrophil‐mediated cell–cell crosstalk. (H) Expression of ligands and receptors in neutrophils.

Next, we investigated cell–cell communication. Both outgoing and incoming signalling revealed the strongest signal from SRCs (Figure 4D, Table S3). The *LAMB3* and *LAMC2*‐mediated (Figure 4E) laminin crosstalk was observed only in SRCs (Figure 4F). Tight communication between neutrophils and other cells was also observed, through the high expression of ligands including *S100A9*, *S100A8* and *CXCL8* and receptors, including *CXCR2* and *CXCR4*.

In conclusion, we found that oncogene amplification during radiotherapy might promote the progression of SRCs, and the activation of Treg cells highly expressed *CTLA4* promote tumour cell survival. The emergence of neutrophils and the activation of macrophages provided an additional immune microenvironment for SRCs. Our findings provide a comprehensive understanding of SRC tumourigenesis, and the activation of CTLA4‐marked Tregs also provides theoretical support for the combination of radiotherapy and anti‐CTLA‐4 therapy.^9^

## AUTHOR CONTRIBUTIONS

Deng Wu, Xishan Wang and Xu Guan conceptualised the study. Deng Wu and Xiaoman Bi performed bioinformatics analysis. Xu Guan and Ran Wei performed histology‐based experiments. Deng Wu, Xu Guan and Xiaoman Bi wrote and discussed the manuscript. Zhixun Zhao, Zhao Lu and Zheng Jiang collected the samples. Deng Wu and Xu Guan supervised the project. All authors have read and approved the final manuscript.

## FUNDING INFORMATION

The work was supported, in part, by grants from the following: The Sanming Project of Medicine in Shenzhen (Ref: SZSM201911012); the National Natural Science Foundation of China (Ref: 82072750); Hainan Provincial Natural Science Foundation of China (Ref: 822QN462 and 823RC581); Youth Science and Technology Talent Innovation Program of Hainan Association for Science and Technology (Ref: QCQTXM202212) and Postdoctoral Science Foundation of Hainan Province.

## CONFLICT OF INTEREST STATEMENT

All authors report there are no potential conflicts of interest.

## ETHICS STATEMENT

This study had been approved by the Ethics Committee of Cancer Hospital, Chinese Academy of Medical Sciences (Approval ID:23/139‐3881)

## Supporting information

Supporting Information

Supporting Information

Supporting Information

Supporting Information

## Data Availability

The raw sequence data reported in this paper have been deposited in the Genome Sequence Archive in the National Genomics Data Center, Beijing Institute of Genomics (China National Center for Bioinformation), Chinese Academy of Sciences, under accession number HRA004786 that is publicly accessible at https://bigd.big.ac.cn/gsa‐human/. Custom codes used to analyse the datasets and generated graphs in the current study are available on GitHub (https://github.com/DBprojects‐lab/SCRectal).
