# Supplementary material for: Single‐cell transcriptomic landscape reveals distinct tumourigenesis and immune microenvironments in secondary radiation‐exposed rectal cancer
Source: Clin Transl Med. 2024 Apr 17;14(4):e1659. doi: 10.1002/ctm2.1659 (PMC11022289; doi:10.1002/ctm2.1659)
Supplement: Supplementary file 1 — Supporting Information [file CTM2-14-e1659-s001.docx]

**Methods**

**Patient recruitment and ethical approval**

In PRC group, patients were diagnosed with first primary rectal adenocarcinoma. Patients in SRC group were diagnosed with secondary rectal adenocarcinoma after the first primary cervical cancer diagnosed and treated with external-beam radiotherapy for the cervical cancer (Figure S1A). Two fresh second rectal tumors and two first primary rectal and adjacent tissues were collected for scRNA-seq. 39 patients with histologically confirmed PRC and 13 patients with SRC were also enrolled in this study, which were prepared from formalin-fixed, paraffin-embedded (FFPE). All clinical samples were collected from the Center for Cancer/Cancer Hospital, Chinese Academy of Medical Sciences and Peking Union Medical College from 2017 to 2018. Written informed consent was obtained from all participants enrolled in this study, and ethical approval was obtained from the following institutional review boards in accordance with the Declaration of Helsinki: National Cancer Center/National Clinical Research Center for Cancer/Cancer Hospital, Chinese Academy of Medical Sciences and Peking Union Medical College.

**Collection of PRC and SRC samples and tissue dissociation**

Fresh rectal tumor and normal tissues were cut into approximately 1 mm pieces in RPMI-1640 medium (Invitrogen) with 10% foetal bovine serum (FBS; ScienCell) and enzymatically digested for 30 minutes on a rotor at 37°C using a MACS tumor dissociation kit (Miltenyi Biotec), according to the manufacturer's instructions. The suspended cells were centrifuged at 400 g for 5 minutes after filtration with a 70 m Cell-Strainer (BD) in RPMI-1640 media (Invitrogen). The pelleted cells were suspended in red blood cell lysis buffer (Solarbio) and incubated on ice for 2 minutes to lyse red blood cells after the supernatant was removed. After washing twice with PBS (Invitrogen), the cell pellets were resuspended in sorting buffer (PBS mixed with 2% FBS). The single cell suspensions were stained for flow cytometry (FACS) on a BD Aria III equipment with 7-AAD Viability Staining Solution (Cat# 00-6993-50, eBiocience). 1 105 live cells were sorted into 1.5 ml tubes with sorting buffer and manually counted under the microscope based on FACS analysis. The GemCode Single Cell Platform was then used to process single cells using the 3' GemCode Gel Bead, Chip, and Library Kits (10 x Genomics) according to the manufacturer's methodology. For each sample, the loaded cell numbers were 10,000. The cells were then partitioned into Emulsion Gel Beads in the GemCode instrument, where they were lysed and barcodes were ligated by reverse transcription; the RNA was then amplified and sheared, and 3' adaptors and sample indexes were ligated. The libraries were sequenced on an Illumina HiSeq 4000 using a 150bp paired-end approach.

**scRNA-seq using 10x Chromium**

The libraries for scRNA-seq were prepared using the Chromium Single Cell 3ʹ v3 according to the manufacturer’s protocol (10x Genomics). A total of 7,000 cells were targeted per sample. Libraries were sequenced on the NextSeq 500 platform (Illumina) with paired-end sequencing.

**Raw data processing and cell type identification**

Raw fastq files were mapped to the reference genome GRCh38 using the Cell Ranger Single Cell (version 5.0.1) software with the default parameters. The filtered results were used as input for the second round of quality control and downstream analysis in Seurat (version 4.0)^1^. To exclude potential cell debris, and doublet from the dataset, we filtered out cells with nFeatrue_RNA ≤200 and ≥7,500. The potential dead cells with high expression of mitochondrial genes (≥25%) were eliminated in the following analysis either. To reduce the potential effect on the cell clustering results, we removed the genes expressed in less than 3 cells, as well as the mitochondrial and ribosome associated genes. The filtered genes were projected into principal component space using the principal component analysis (RunPCA). The top significant 50 dimensions identified by ElbowPlot were used graph-based clustering. t-distributed stochastic neighbour embedding (t-SNE) was utilized in dimensionality reduction of cells for visualizing, and shared nearest neighbor-based clustering algorithm was used to find the clusters. FindAllMarkers was used to identify the cell type specific genes with the parameters logfc.threshold = 0.25, only.pos = TRUE, and min.pct = 0.25. By integrating the cell type specific markers identified in previous studies^2, 3^, validated cell makers curated in CellMarker^4^ and the cell type specific expressed genes, we assigned a cell type identity to each cell cluster.

**Cell type-specific transcriptomic changes**

FindMarker function was used to examine the cell type (clusters) changes in primary and second rectal cancer with the min.pct = 0.25, logfc.threshold = 0.25, and test.use = "wilcox". The genes with p_val_adj≤0.01 were selected as the significantly differentially expressed genes (DEGs). The clusterProfiler package was used to evaluate the over-representation of DEGs in each cluster.

**Subcluster analysis**

To further analysis the certain cell types at a higher resolution, we are using the subset function to isolate the target cell type from the original Seurat dataset by their cell type name. And then a similar approach to initial cell type clustering was used to re-cluster each cell type. As the different complexity of cell type, various principal components and resolutions were used. By utilization of ElbowPlot and clustree we selected the top 50, 20, 20, and 50 dimensions and resolution with 0.7, 0.5, 0.5, and 0.1 as input to find subcluster of epithelial cells, T cell, myeloid cells, and B cells, respectively.

**Identifying neoplastic from normal rectal epithelial cells**

To distinguish the neoplastic from normal cells, copy number variations for each cell were estimated using the R package inferCNV (https://github.com/broadinstitute/inferCNV). The sub epithelial clusters with a small percent of rectal cancer patients were used to define the reference. The cutoff was set as 0.1 to remove the genes expressed less than 10 percent cells, and denoise as true to obtain the significant copy number variation signal.

**Cell cycle scoring of epithelial cells**

Previously reported cell cycle associated genes and their corresponding phase information were extracted from both the Whitfield et al and Tirosh et al studies. Within this combined list, only genes detected by single cell sequencing were retained. The scoring function AddModuleScore with default parameters from the Seurat package was used to calculate a cell cycle phase specific score for each cell. The variation between normal, PRCs and SRCs were obtained via Student's t test.

**Analysis of cell-cell communications in the rectal cancers**

CellChat^5^ was used to investigate the cell-cell communication variation in both the primary and second rectal cancers. The epithelial cells and the immune response associated cells were isolated to decipher their potential cell-cell communications. To build a more comprehensive cell-cell communication pathway, we projected the ligand-receptor interactions from CellTalkDB^6^ into CellChat as the background. Considering the variation in cellular composition among patients, we performed the cell-cell communication analysis by segregating cells from each patient. **Multiplex Immunofluorescence Staining**

We used the Opal-7-Color Manual IHC Kit (211011069; Panovue, Beijing, China) and the Tyramide Signal Amplification Fluorescence Kit (Panovue, Beijing, China) to assess the expression and distribution of gene related to tumor microenvironment. Each of these tissues was cut into pieces of 1.0 mm and attached to the slides (5 mm thick) from the FFPEs. The FFPEs were incubated with Anti-CD3 (ab5690; 1:100; Abcam, Cambridge, United Kingdom), Anti-CD4 (ab133616; 1:100; Abcam, Cambridge, United Kingdom), Anti-CD8 (ab237709; 1:100; Abcam, Cambridge, United Kingdom), Anti-BCA1 (ab199043; 1:100; Abcam, Cambridge, United Kingdom), Anti-CTLA4 (ab237712; 1:100; Abcam, Cambridge, United Kingdom) and Anti-CD127 (ab259806; 1:100; Abcam, Cambridge, United Kingdom) antibodies at 4°C overnight, and then with horseradish peroxidase-conjugated secondary antibody and tyramide. A microwave was used to heat-regenerate the TMAs after each Tyramide Signal Amplification step. We used 4’,6-diamidino-2-phenylindole (DAPI) to counterstain the cell nuclei.

**Proteins quantification from immunofluorescence staining images**

The Mantra System (PerkinElmer, Waltham, Massachusetts, US) was used to capture the multispectral immunofluorescence images with the fluorescence spectra at 20-nm wavelength intervals from 420 to 720 nm with the same exposure time, which were then composited to establish a single stack image. To capture images of sections without autofluorescence, we extracted the spectrum of autofluorescence of TMAs and each fluorescein from the images of unstained and single-stained sections, which were used to establish the spectral library for multispectral unmixing using inForm image analysis software (PerkinElmer, Waltham, Massachusetts, US). Two independent pathologists analyzed and counted single-positive cells and the expression of these genes in each tissue at 200× magnification in a blinded manner. The nucleated stained cells were quantified and expressed as the number of cells. The positive rate of single index and single index intensity score were employed to evaluate the expression and distribution of the identified genes in cancer, which were calculated by multiplication of the multiplex immunofluorescence staining intensity (percentage of single index %= Number of positive cells/ Total number of cells; The 25% staining was taken as the threshold of the strength score; 25–49%, strength I; 50–74%, strength II; 75–100%, strength III; single index strength score=[(strength I*positive rate of single index) *1+(strength II*positive rate of single index) *2+(strength III*positive rate of single index) *3] *100).

**Transcription factors identification for tumor cells**
Transcription factors for each subtype of epithelial cells were identified by SCENIC packages^7^. The transcription factors with auc score in each cluster of epithelial cells were calculated to evaluate their potential regulation effect. The cisTarget database used in this part includes hg38__refseq-r80__10kb_up_and_down_tss. Feather and hg38__refseq-r80__500bp_up_and_100bp_down_tss.mc9nr. Feather was downloaded from https://resources.aertslab.org/cistarget/.

**Data and code availability statement**

The raw sequence data reported in this paper have been deposited in the Genome Sequence Archive in National Genomics Data Center, Beijing Institute of Genomics (China National Center for Bioinformation), Chinese Academy of Sciences, under accession number HRA004786 that is publicly accessible at <https://bigd.big.ac.cn/gsa-human/>. Custom codes used to analyse the datasets and generated graphs in the current study is available on GitHub (https://github.com/DBprojects-lab/SCRectal).

1. Hao, Y. *et al.* Integrated analysis of multimodal single-cell data. *Cell* **184**, 3573-3587 e3529 (2021).

2. Che, L.H. *et al.* A single-cell atlas of liver metastases of colorectal cancer reveals reprogramming of the tumor microenvironment in response to preoperative chemotherapy. *Cell Discov* **7**, 80 (2021).

3. Pelka, K. *et al.* Spatially organized multicellular immune hubs in human colorectal cancer. *Cell* **184**, 4734-4752 e4720 (2021).

4. Hu, C. *et al.* CellMarker 2.0: an updated database of manually curated cell markers in human/mouse and web tools based on scRNA-seq data. *Nucleic Acids Res* **51**, D870-D876 (2023).

5. Jin, S. *et al.* Inference and analysis of cell-cell communication using CellChat. *Nat Commun* **12**, 1088 (2021).

6. Shao, X. *et al.* CellTalkDB: a manually curated database of ligand-receptor interactions in humans and mice. *Brief Bioinform* **22** (2021).

7. Aibar, S. *et al.* SCENIC: single-cell regulatory network inference and clustering. *Nat Methods* **14**, 1083-1086 (2017).

**Supplementary figures**


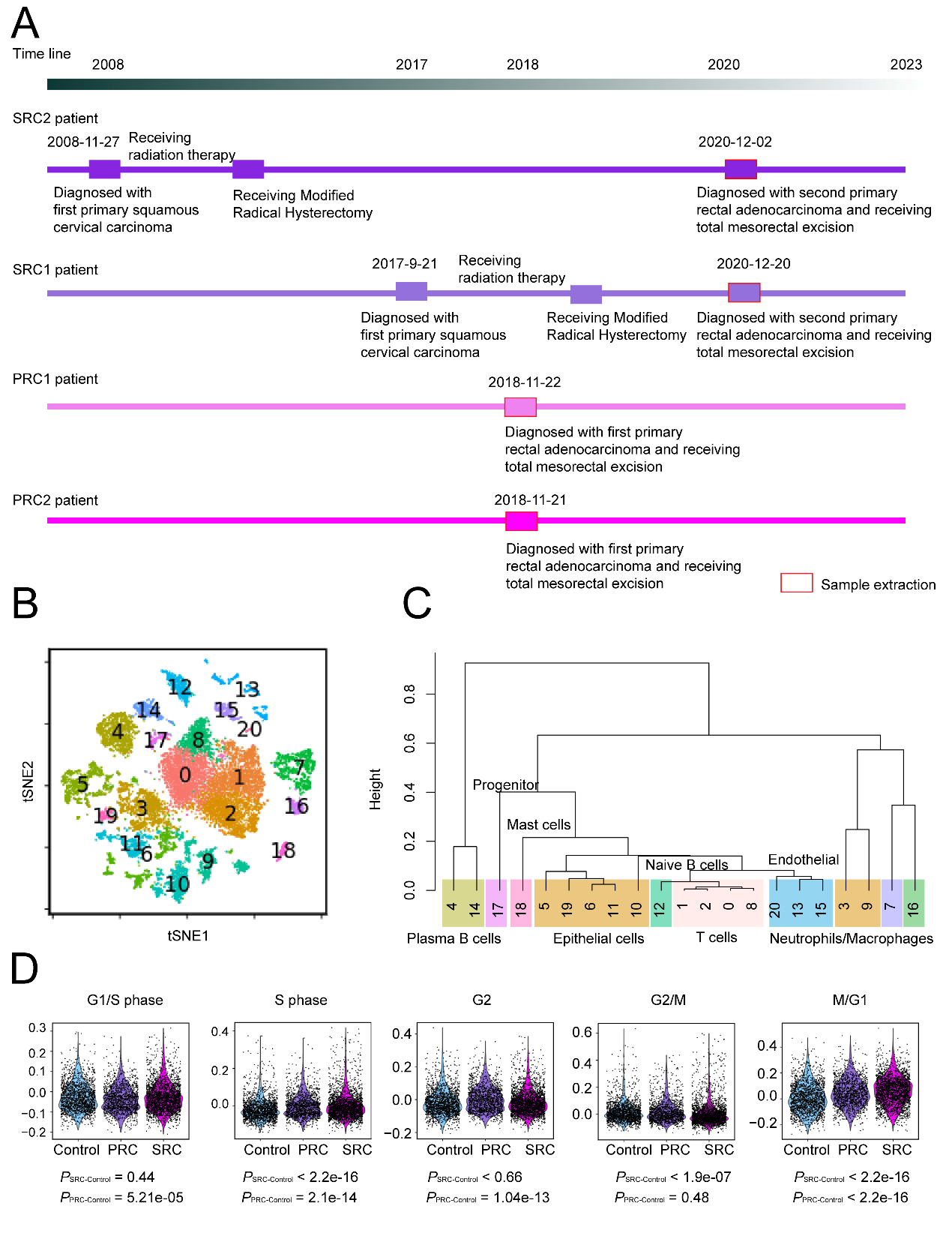


Figure S1. Cell type identification across the primary and second rectal cancers. A, Identification of the secondary rectal cancers (SRCs) according to their clinical phenotypes. B, tSNE visualization of cell clusters. C, unsupervised clustering of the cell clusters. D, boxplots of the cell cycle score between control, PRCs and SRCs. M to G1 phase showed significantly increased in both PRCs and SRCs.


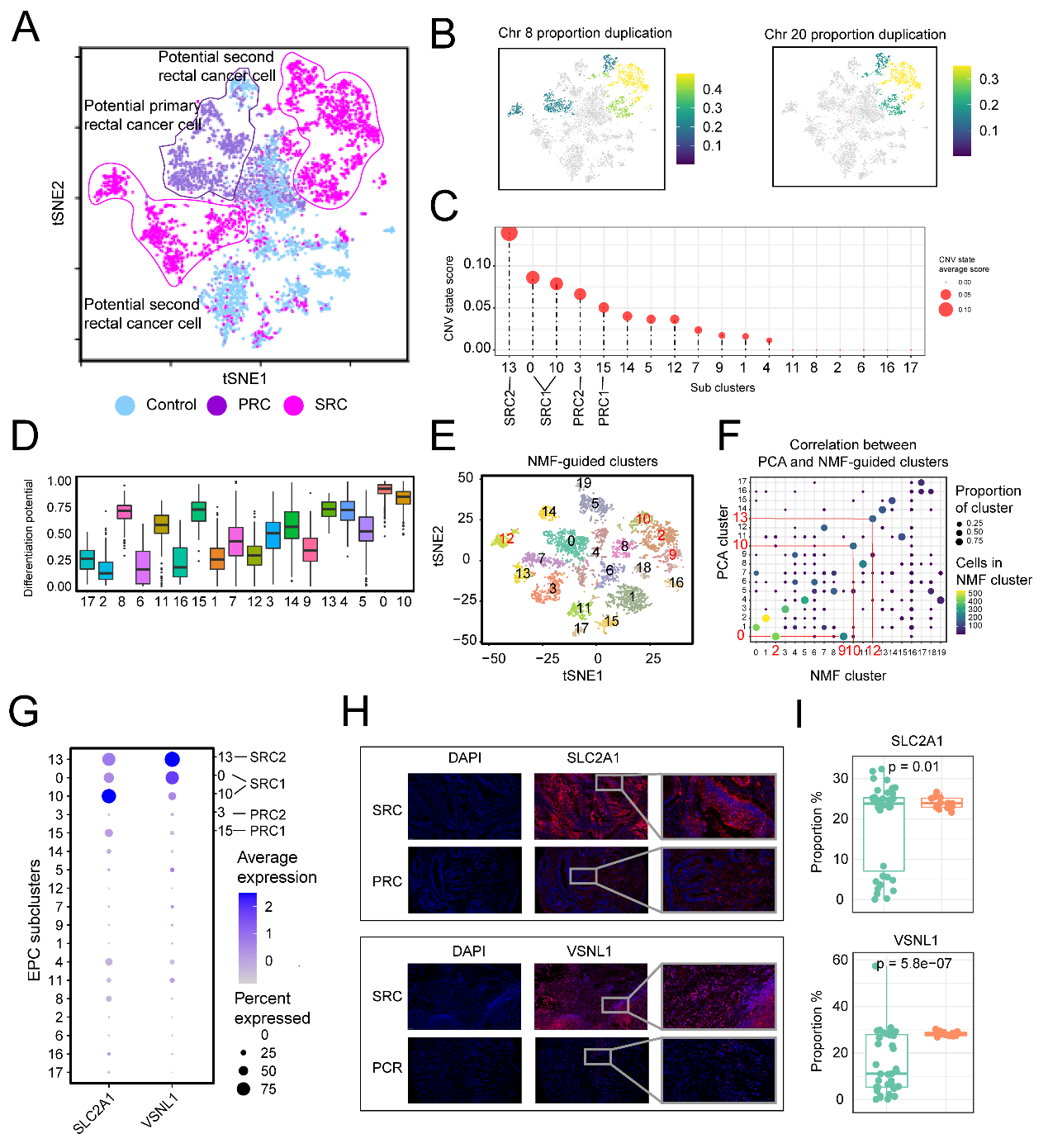


Figure S2. Landscape of epithelial in rectal. A, tSNE plot for the sample group, B, tSNE plot of the quantification of the gain of chr 8 and chr 20. C, Lollipop plot of the average CNV state score for each subcluster. D, potential differentiation ability which was estimated via SCENT for each cluster. E, tSNE plot of epithelial cells which was generated by non-negative matrix factorization (NMF)-dependent dimensionality reduction method. The clusters with red labels represented the tumor cells from SRCs which identified by PCA-dependent dimensionality reduction. F, Correlation quantification between NMF- and PCA-guided clusters. The tumor cells including cluster 0, 10, and 13 which were identified by PCA perfectly matched to the cluster 2, 9, 10, 12 which were generated by NMF method. G, Definition of SRC malignant cell-associated markers according to their expression levels between subclusters of EPCs. We prefer to Cluster 13, 0, and 10 represented the malignant cells of SRCs and SLC2A1 and VSNL1 were selected as the markers of SRC malignant cells which specifically highly expressed in these subclusters. H, immunofluorescence staining images of the expression of SLC2A1 and VSNL1 between PRCs and SRCs. I, quantification of the expression of SLC2A1 and VSNL1 in additional samples. The p value was conducted using t test.


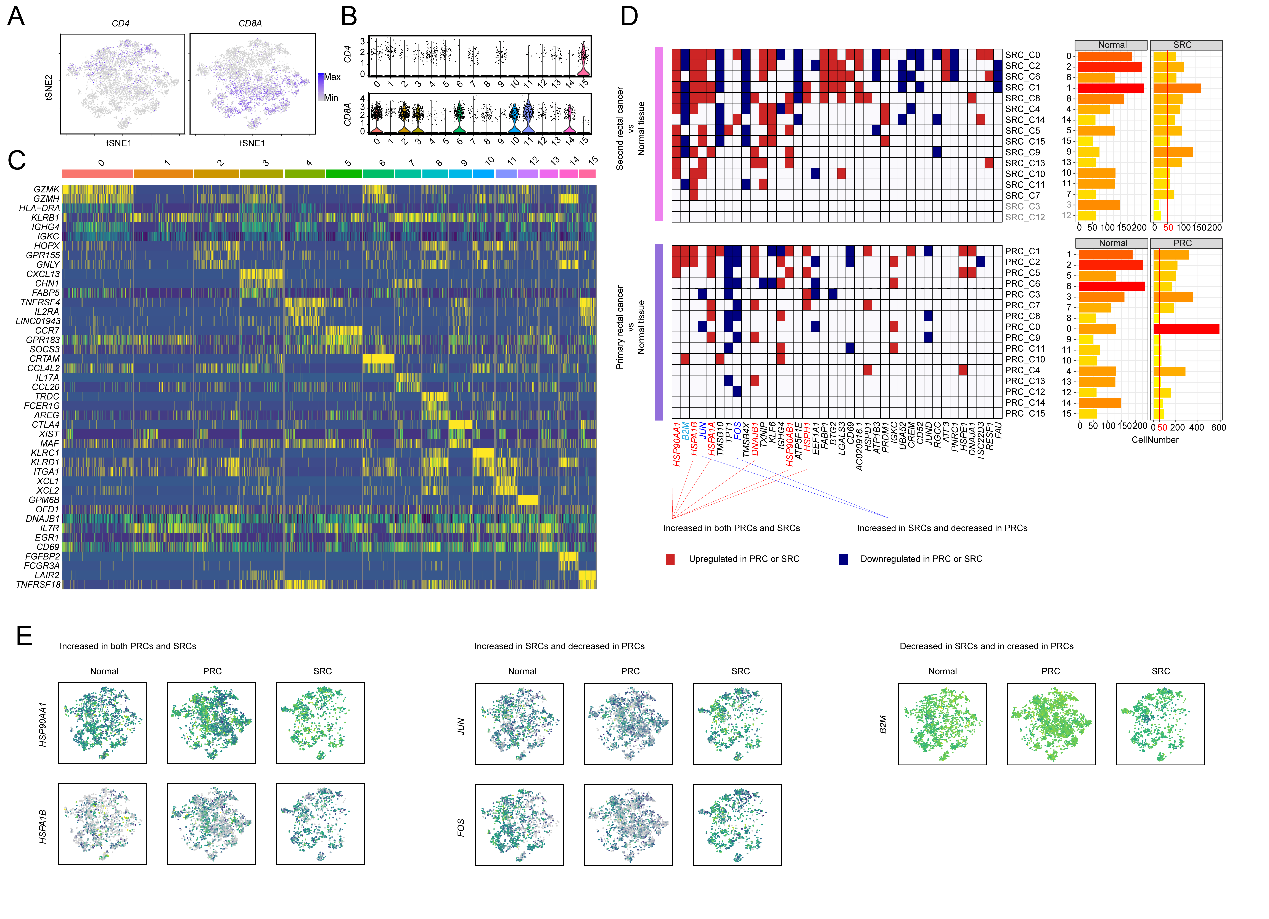


Figure S3. Markers for each subtype of T cells. A-B, expression of CD4 and CD8 in each cluster for T cells to identify the CD4 and CD8 positive T cells. C, expression of the sub-cluster specific marker genes in T cells. D, DEGs between tumors and normal cells within each cluster were shown in left. The blue squares represent the downregulated genes, and the red squares represent the upregulated genes in tumor cells. The total number of cells in each cluster are shown in the right panel. Sub-cluster 3 and 12 were not considered in identification of DEGs as the limited cells (<50 cells). E, tSNE plots displayed the expression of DEGs from D. Left two genes represent the DEGs increased in both PRCs and SRCs, middle panel represent the DEGs decreased in PRCs and increased in SRCs, and the right panel represents the DEGs increased in RPCs and downregulated in SRCs.


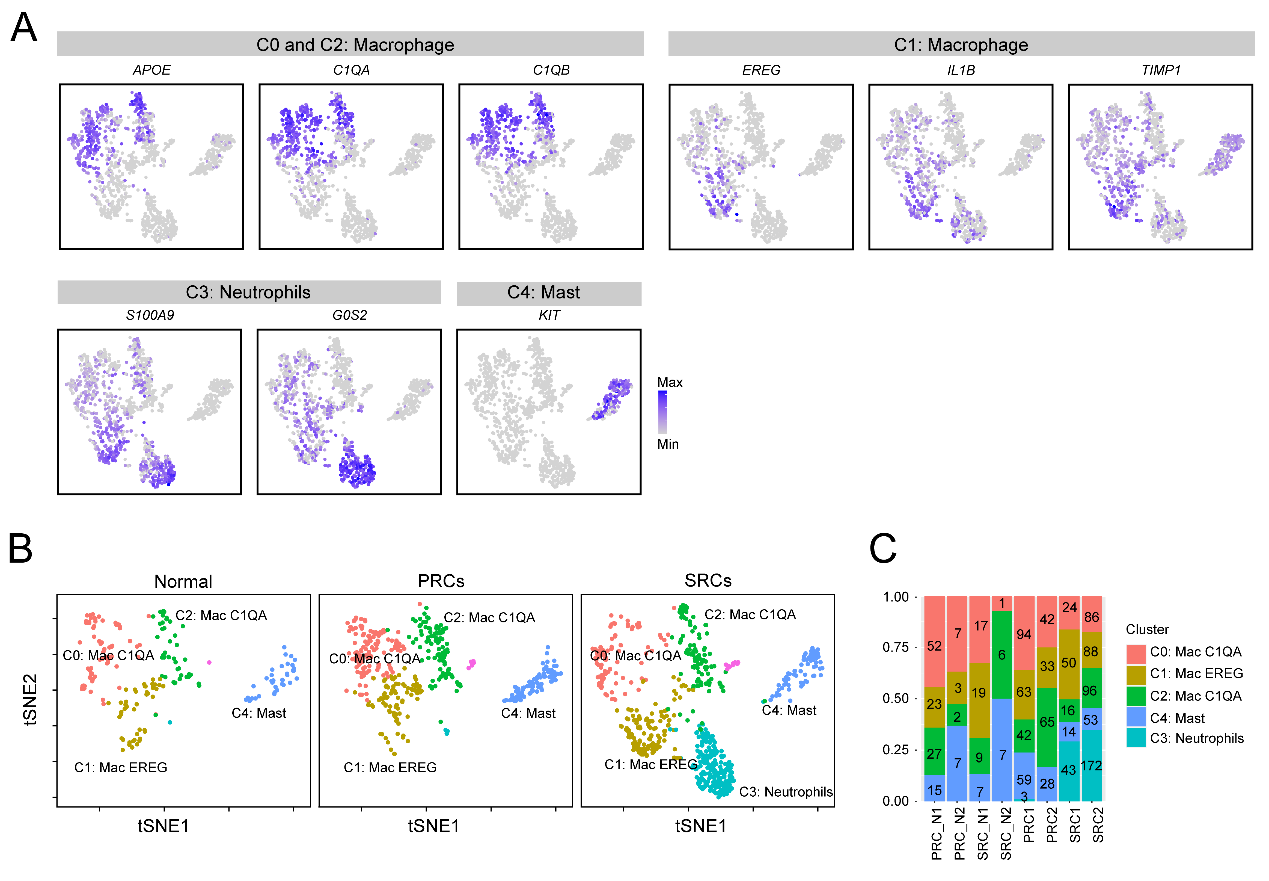


Figure S4. Cell markers for myeloid cells. A, expression of the sub-clusters of myeloid cells associated markers. B, tSNE plots for the myeloid cells between normal, PRCs, and SRCs. C, The total number of of cells within each sub-cluster in every sample.
